# Development and initial validation of a dual-purpose questionnaire capturing mentors’ and mentees’ perceptions and expectations of the mentoring process

**DOI:** 10.1186/s12909-019-1574-2

**Published:** 2019-05-08

**Authors:** Sylvia Heeneman, Willem de Grave

**Affiliations:** 1 Department of Pathology, Faculty of Health, Medicine and Life Sciences, Maastricht University/ MUMC, Peter Debyelaan 25, Maastricht, HX 6229 The Netherlands; 2 Department of Educational Development and Research, Faculty of Health, Medicine and Life Sciences, Maastricht University, Universiteitssingel 60, Maastricht, ER 6229 The Netherlands

**Keywords:** Mentor, Mentor-student relationship, Needs assessment, Portfolio

## Abstract

**Background:**

In health profession education, learners are often coached by mentors for development of competencies, self-direction of learning and professionalism. It is important that the mentee-mentor relationship is aligned in terms of mutual expectations.

**Methods:**

A dual-purpose questionnaire capturing both the mentor and mentee perceptions on the actual and preferred mentoring functions was designed and validated, by performing a principal component analysis (PCA) using the data of mentees (*n* = 103) and mentors (*n* = 23) of a medical course. As a proof of concept, alignment of needs and changes in the mentoring perceptions in mentee groups of different years were determined.

**Results:**

PCA showed that specific sets of questions addressed important elements in the mentoring process, such as self-direction of learning and reflection (Scale 1), guidance of behavioural change (Scale 4), addressing personal issues and professional identity development (Scale 3 and 5) and how the mentor and mentee presents oneself in the mentoring relationship (Scale 2). Mentors and mentees perceived comparable situations as critical for an effective mentoring process, such as mentor presence and guidance of reflection, although there was also evidence of gaps, such as perception of cultural issues. By comparison of the mentee groups in the different years of the program, the dynamic or evolving nature of the mentor process became evident, mentees experienced more emphasis by the mentor on reflection (Scale 1), at a constant level of mentor presence (Scale 2).

**Conclusion:**

Given the individualized, context-specific, and dynamic nature of mentoring, programmes would benefit from a regular evaluation of mentoring practices, e.g. by using questionnaires, in order to facilitate organizational revisions and further development of the mentoring competencies.

## Background

Mentoring is perceived as an important element of health profession education. Although varying definitions are used, and mentoring is often situational and context-specific, recurrent components of a mentoring relationship in health profession education are support and guidance for the mentees’ professional and personal development, by a faculty member or a peer, through the sharing of experiences, knowledge, skills, and wisdom [1, 2]. There is general consensus that mentoring programmes have beneficial effects on job satisfaction, career success, personal development, reflection and self-direction of learning. These benefits have been described for the mentee and for the mentor [1–7]. However, negative experiences have also been described, such as a low quality of the relation or dominance of the mentor, and have been called the ‘dark side’ of mentoring [8], which affects both the mentor and mentee. The underlying causes for negative mentoring experiences are likely to be multidimensional and can lie in the domain of the mentee, in terms of lack of trust, or bad intent in the relationship, or can be at the side of the mentor, in terms of dysfunctional behaviour, due to lack or insufficient training, or the inability to adapt to changing needs of the mentee [9, 10]. The latter is important as increasingly mentor relationships are long-term, spanning the length of the program, using tools such as a portfolio in the context of some form of assessment [11–13], especially in health profession education such as nursing and medical education. In addition, it was shown that satisfaction in the mentoring process was the best predictor for a successful relationship in the long term [14]. These studies show that it is extremely important that expectations of mentors and mentees on the mentoring process are aligned and especially that the mentor can adapt to the needs of the mentee during different phases of the education. There is limited information how to evaluate and subsequently guide and support alignment and mutual expectations in the mentoring process.

In terms of evaluation of the mentoring process, several mentorship measurement scales have been described. In a recent literature review by Chen et al. [15], 22 scales for measuring mentors’ function, behaviour and relationships were found. It was shown that scales from the field of business and organisation are founded in a common theoretical framework with a 3-function structure: career, psychosocial, and role modelling function. This was resonated in scales such as the Mentorship Relationship Challenges Scale and the Mentoring Functions Questionnaire - 9 [16, 17]. In these questionnaires, career support is represented as a personal interest of the mentor in career and coordination of professional goals. Psychosocial support is represented by a safe environment in which the mentee can share personal problems and exchange confidences. In addition, the mentor is an example for behaviour and is respected for his/her ability to motivate and teach others. In the health profession educational field however, no universal recognised framework for mentoring was yet found, implying that in education, mentoring is conceptualized context-dependently [18], and used in varying situations, also suggesting that more measurement tools will need to be developed [15]. Recently Schäfer et al. [19] developed a questionnaire for evaluating mentees’ satisfaction with the mentoring relationship in the medical education field. Although addressing an important determinant of a successful mentoring relationship in the long term; the satisfaction in the relationship, this questionnaire addressed the mentees’ needs and expectations, while it is equally important to get insight in the mentors’ perspective and needs, to align mutual expectations and guide mentoring support.

In their review about available mentorship measurement tools, Chen et al. [15] also noted that there was a trend in scale development from focusing on the positive aspects of mentoring to (negative) mentoring experiences and challenges [16, 20]. Stories of critical incidents by mentors also revealed that challenges such as a mentee resisting mentoring or not attending meetings led to a dynamic nature of the mentoring process and fluctuations in reasoning and behaviour of the mentor [21]. In a previous study, we interviewed mentors in a medical education program and also found that student-centred critical incidents, such as students with poor reflective, self-directed learning styles or resenting the use of the portfolio, were perceived as a challenge, as well as an opportunity for professional development [12]. Here, a critical incident is the narrative of a challenge, a situation or behaviour that is perceived as potential risk for an effective mentor meeting [22]. Mentoring success is critically dependent on an effective relationship that is characterized by a safe setting and a nurturing and trusting personal connection [18], which could be hampered by critical incidents. For an effective relationship, it is vital that there is a congruency between the expectations and actual experiences of critical incidents for both mentors and mentees. In addition, mentoring is a dynamic and developmental process in which the relationship and interaction between mentor and mentee change over time [23]. Therefore, there is a need for an instrument that can evaluate the dual perspective and level of alignment. The aim of the current study was the development and initial validation of a dual-purpose questionnaire capturing both the mentor and mentee perceptions on the actual and preferred mentoring functions and critical incidents. As a proof of concept, the questionnaire was distributed amongst mentees and mentors of a medical course to perform a needs analysis and to determine whether use of the questionnaire would demonstrate important gaps or misalignments in the mentoring process. This study was done in the context of a longitudinal mentoring programme, in which a portfolio was used for self-regulation of learning (by the mentee), and guidance (by the mentor). The portfolio was assessed at the end of each year. The research questions were: (i) What are the underlying dimensions of the questionnaire? (ii) What are critical incidents in the mentoring process according to mentees and mentors and what is the level of alignment? And (iii) What are the perceptions of mentees on actual and preferred mentoring function in different years of the program?

## Methods

### Setting and participants

This study was performed at Maastricht University, the Netherlands. The participants were mentees and mentors in the graduate-entry program Physician-Clinical Investigator, which is a 4 Year medical course. The set-up of the medical program and mentorship program is described in Heeneman and de Grave [12]. Briefly, the distinguishing features of this medical course are the use of programmatic assessment, and a longitudinal mentoring relationship during the entire course. The assessment of the course as a whole is designed according to the principles of programmatic assessment [24, 25]. All assessment and feedback information is collected and aggregated in an electronic portfolio. Students use all assessment and feedback information that the program generates, to self-regulate their learning, in a regular process of reflection captured in the portfolio, which is used in the mentor meetings throughout the years. Students are appointed the same mentor for 4 years. Mentors meet with their students 5 times in Year 1 (39 weeks of pre-clinical education according to the principles of problem-based learning), 3 times in Year 2 (27 weeks, patient-based preclinical education), 4 times in Year 3 (64 weeks, clinical placements/ clerkships) and 2 times in Year 4 (30 weeks, senior elective clinical placement combining clinical care and translational research). The topics of the mentor meetings are not strictly defined (i.e. no set agenda for each meeting) and the content might vary depending on the questions or topics of both the mentee and the mentor. Topics that are regularly discussed are the performance of the student (based on the assessment and feedback information in the portfolio), the subsequent analysis and reflection and follow-up of learning goals, choices that the mentee can make in the curriculum (e.g. content and topic of electives, vocational interest, career choices), personal development, and personal problems. At the end of the Year, the portfolio is assessed by an independent portfolio assessment committee, leading to a high-stake (pass or fail) promotion decision to the next year or graduation. The mentors are faculty members with a PhD degree, working as a clinician-investigator in the university hospital or as an investigator/ scientist in a research group of the university. Every mentor has 4–10 students, with no more than 5 students in the same year group. There is a yearly training in coaching and interviewing techniques at the beginning of the year, that all mentors attend. During the year, there are 5 mentor meetings, in which a topic (e.g. how to address certain assessment results) is discussed and there is opportunity to exchange experiences and sharing of stories.

### Design of the questionnaire

The questionnaire was developed using the results of an earlier qualitative interview study with the mentors [12] and mentoring dimensions found in the literature [1, 3, 5, 11, 23, 26–33]. Mentoring dimensions included were performance indicators and mentees analysis thereof, choices to be made in curriculum (e.g. electives, research opportunities), vocational interests/ career planning, and personal development and wellness. This resonated with the critical situations as perceived by the mentors concerning the mentees, and the critical incidents that the mentors perceived in their own behaviour and function, and the combination led to the construction of a 38 item questionnaire, in an edition to be used for mentors and an edition to be used for students (see Table 1). The items were verbalised as critical situations in the mentor version and in terms of mentoring functions or behaviours in the mentee version, e.g. ‘*The mentee has difficulties translating feedback and assessment information for certain competencies (such as the health advocate) into learning objectives*’ (Mentor version) or *‘The mentor helps me to formulate learning objectives in competency domains that are rather thorny (health advocate, for example)’* (Mentee version). For each critical situation the respondent indicated in a Likert scale 1–5 whether

1. The situation was *experienced* in a mentor meeting (1 = never, 5 = at multiple occasions/ repeatedly), as an indication if the situation or critical incident was (actually) experienced (by mentor and mentee);
2. The situation *prevents* the mentor meeting from *being effective* (*mentor version*), or in the mentee version: This situation is *important* for *an effective mentor meeting* (1 = disagree, 5 = totally agree) as an indication of what mentor behaviour or function was disruptive or wanted;
3. It is *mentors’ responsibility/ job to take action* when this situation occurs (1 = disagree, not mentors’ responsibility, 3 = sometimes, depends on the situation, 5 = agree, this should always be discussed, the mentor should act), as an indication of what mentor behaviour or function was perceived as important by mentor or mentee.

The questionnaire ended with one open question in which the respondents could indicate whether situations that they perceived as important or critical were not present in the items of the questionnaire.

As a pilot, the questionnaire was completed by *n* = 3 experienced mentors and *n* = 6 mentees and their feedback was used in the final version of the questionnaire.

**Table 1:** Principal component analysis of the mentees’ responses (*n* = 103), factor loadings after rotation, scales and reliability (Cronbach’s α)

ITEM (mentee-version of the questionnaire) and SCALES	1	2	3	4	5
SCALE 1: Mentor stimulation of reflection; Cronbach’s α .81					
Situation 13. The mentor challenges me to deepen my reflection	.713				
Situation 8. The mentor encourages me to analyse my self-image critically.	.697				
Situation 7. During the progress meetings, the mentor provides feedback about my progress in the competencies and level of reflection	.643				
Situation 18. The mentor encourages open discussion of my personality traits (such as perfectionism; dominant behaviour).	.613				
Situation 4. The mentor challenges me to go beyond the borders of my (sufficient/good) current performance.	.596				
Situation 6. The mentor pays sufficient attention to the discussion of all key and enabling competencies.	.557				
Situation 12. The mentor encourages me to reflect on specific personal experiences.	.544				
Situation 28. The mentor encourages me to reflect on my development (through placements, over the years).	.464				
Situation 23. The mentor especially considers the mentor meeting as an assessment moment, rather than a moment of support.	−.415				
SCALE 2: Mentor presence; Cronbach’s α .77					
Situation 30. The mentor shares his or her thoughts and feelings about incidents that occur during the mentor meetings.		.702			
Situation 31. The mentor pays attention to my emotional experiences (in workplace/private life).		.669			
Situation 36. The mentor shares his or her experiences with me during meetings.		.618			
Situation 38. The mentor discusses my insecurities about the portfolio assessment/assessment feedback.		.563			
Situation 35. The mentor is readily available for contact		.551			
Situation 1. The mentor exhibits professional behaviour. For instance: the mentor came prepared to each meeting; the mentor keeps his/her engagements.		.550			
Situation 9. The mentor tries to build a good rapport with me		.501			
SCALE 3: Mentor addressing personal issues; Cronbach’s α .80					
Situation 32. The mentor pays attention to my incentives to study (e.g., motivational ups and downs).			.625		
Situation 25. The mentor discusses my experiences with planning and time management.			.615		
Situation 34. The mentor supported me in forging new, relevant contacts for study and career choice-related purposes.			.609		
Situation 33. The mentor thoroughly discusses lows/ problems with regard to my study progress.			.605		
Situation 21. The mentor discusses the balance between my studies and private life with me.			.552		
Situation 24. The mentor discusses with me my insecuri-ties with respect to the choices I need to make about studies/career.			.514		
Situation 2. The mentor encourages me to learn pro-actively in the workplace/ during study activities.			.426		
Situation 3. The mentor is focused not only on the portfolio, but also on the discussion of personal experiences.			.421		
Situation 17. The mentor encourages open discussion of my personal experiences affecting my studies.			.461		
SCALE 4: Mentor stimulating conditions and perspectives for behavioural change; Cronbach’s α .79					
Situation 10. The mentor helps me to formulate learning objectives in competency domains that are rather thorny (health advocate, for example).				.632	
Situation 5. The mentor discusses how I should treat confidential information about the workplace with care.				.599	
Situation 29. The mentor discusses how he or she will treat confidential information about me.				.598	
Situation 16. The mentor follows up on the attainment of my predefined learning objectives.				.582	
Situation 15. The mentor discusses with me how I can pursue my learning objectives in practice.				.512	
Situation 14. The mentor challenges me to formulate specific and attainable learning objectives.				.497	
Situation 11. The mentor discusses and analyses the results of the progress test with me.				.459	
SCALE 5: Mentor addressing professional identity development; Cronbach’s α .64					
Situation 26. The mentor challenges me to avail myself of/ use my own qualities, values and strengths.					.680
Situation 19. The mentor discusses with me how I envision the development of my identity into a doctor.					.535
Items not loaded or omitted after analysis					
Situation 20. The mentor discusses the importance of the portfolio with me.					
Situation 22. For the most part of the meetings the mentor is assuming a guiding role (giving suggestions and advice)					
Situation 27 – The mentor clearly sets the boundaries of his or her support (for instance by means of referral)					
Situation 37 – The mentor is sensitive to my (cultural) background					

### Data collection

The questionnaires were distributed to students at the end of Year 1 (*n* = 38, response rate 76%), end of Year 2 (*n* = 31, response rate 62%) and end of Year 3 (*n* = 33, response rate 66%), and to the mentors that completed at least 1 year of mentoring (*n* = 20, response rate 67%). Response rates were considered acceptable. The questionnaires were distributed and immediately collected in plenary sessions/ lectures; the non-responders were students or mentors that did not attend these sessions.

### Statistical analysis

To evaluate whether potential constructs or scales could be identified in the questionnaire, principal component analysis (PCA) was used on the collective data of the mentees (*n* = 103) [34]. Cut-off for item loading was set at 0.400. After PCA, average scale scores were generated for the sets of questions that showed component loading on the different scale themes. Cronbach’s Alpha for these average scale themes were calculated to assess whether reliability of the sub-scales was sufficient.

Descriptive statistics (mean ± standard error of the mean (sem)) were used to determine the highest scoring situations from the mentees’ (*n* = 103) and mentors’ (*n* = 20) perspective. Item 23 and 27 were reversed before calculation given the negative factor loadings and negative corrected item-total correlation values in the item-total statistics in the reliability statistics.

To get an impression of the gap between the actual experienced situation (question 1) and what was deemed important for an efficient meeting (question 2), the individual value of question 1 was distracted from the value of question 2. A positive difference was used as an indication of the gap, then what was wanted or deemed important, did not align with what was experienced.

One-way ANOVA with post-hoc Bonferroni testing was used to determine which situations of actual and preferred mentoring functions were distinctive in the mentee groups of different years.

#### Analysis of open-ended question

The remarks were read and aligned according to similar themes and the themes that were mentioned most frequently were reported.

## Results

### Identified scales in the questionnaire

A PCA was done on the 38 items of the questionnaire, using the responses of the mentees (*n* = 103) for the question 1: The situation was experienced in a mentor meeting. The rationale was that this question expressed the need and expectations of the mentee. The KMO measure verified the sampling adequacy for the analysis, KMO = .693 (above the minimum of .5). The Bartlett’s test of sphericity confirmed that the original correlation matrix was an identity matrix (Chi-square = 1513, df = 703, *p* < .001) [34]. An initial analysis was done to obtain eigenvalues for each component in the data. Eleven components had eigenvalues over 1 and in combination explained 69.6% of the variance. The scree plot was ambiguous and showed inflexions that would justify retaining less components (not shown). A PCA was performed with 4, 5 and 6 factors, using a Varimax rotation, the results compared and assessed for best fit. Given a certain subjectivity and considering the sample size, a rotated solution with 5 factors was considered to produce an acceptable fit and factorial suitability, both intuitively and conceptually. The five factors explained 48% of variance. Table 1 shows the factor loadings after rotation. Two items did not show sufficient factor loading (< 0.400) to any of the scales (item 20, 22).

Regarding the representation of the scales, the items that clustered on factor 1 were on the stimulation of reflection and challenging the mentee suggesting that Scale 1 represented ‘*Mentor stimulation of reflection*’ (*n* = 9 items). In Scale 2, mentor behavior was represented, such as sharing of personal experiences, and being attentive to a good relationship, therefore Scale 2 was indicated as ‘*Mentor presence*’ (*n* = 7 items). In Scale 3, the items focused on personal issues of the mentee, such as motivation, proactive behavior and planning, which was summarized as ‘*Mentor addressing personal issues*’ (*n* = 9 items). In Scale 4, situations revolved around behavioral change through formulation and follow-up of learning goals, Scale 4 was summarized as ‘*Mentor stimulating conditions and perspectives for behavioral change*’ (*n* = 7 items). Scale 5 was relatively small (4 items) and addressed the use of qualities and the identity as a medical doctor, and was designated as ‘*Mentor addressing professional identity development’*.

The overall Cronbach’s α of the 38 item questionnaire was .90. The reliability of Scale 1–4 was sufficient, with Cronbach’s α between .77 and .81 (Scale 1 α = .81, Scale 2 α = .77, Scale 3 α = .80, Scale 4 α = .79). In Scale 5, two items affected reliability (item 27 and 37). After deletion, reliability of this scale, Cronbach’s α .64, was lower as compared to the other scales. Although the correlation between the 2 remaining items (19 and 26) was sufficient (.51), this scale, containing 2 items, has to be viewed with some caution.

The responses on the open question on situations that were missed confirmed content validity, as analysis of the answers yielded few omissions. Mentioned were: a lack of items on the portfolio itself from the mentees (as the portfolio is the aggregation instrument, we choose to focus on the content), on a context-specific procedure in the mentoring process of this program (from mentees and mentors), and on the discussion of professionalism issues such as fraud and plagiarism (from mentors).

### Critical incidents in the mentoring process according to mentees and mentors and level of alignment

In Table 2a, an overview of high scoring situations or incidents is shown (question 1). The mentee clearly experienced from their mentors a personal approach (Scale 2, Mentor presence), a coaching behaviour on the reflective activities (Scale 1, Mentor stimulation of reflection) and addressing personal issues (Scale 3, Mentor addressing personal issues). This was also seen in the mentor perception and then formulated as critical incident in either reflection, personal issues (Scale 1 and 3) or the mentee (not) sharing their thoughts (Scale 2, Mentee presence, in the perspective of the mentor). However, mentors also put emphasis on the support of behavioural change (Scale 4, Mentor stimulating conditions and perspectives for behavioural change), this was presented more often in the Top 10 of situations from mentors.

**Table 2:** Overview (Top 10) of high scoring situations that were experienced (Table 2a: Question 1) and perception of the mentors’ tasks or responsibilities (Table 2b: Question 3)

	Mentee (*n* = 103)	Mentor (*n* = 20)
A. The situation was experienced in a mentor meeting (1 = never, 5 = at multiple occasions/ repeatedly), as an indication if the situation or critical incident was (actually) experienced. In brackets are the mean ± sem).		
1	Situation 1 – The mentor exhibits professional behaviour (4.37 ± 0.07) *Scale 2: Mentor presence*	Situation 10 – The mentee has difficulty translating a specific key or enabling competency (such as health advocate) into learning objectives (4.40 ± 0.13) *(Scale 4)*
2	Situation 23 – The mentor especially considers the mentor meeting *(‘not’)* as an assessment moment, rather than a moment of support (4.30 ± 0.09) > Reversed calculation given the negative verbalization *Scale 1: Mentor stimulation of reflection*	Situation 13 – Reflections by the mentee fall short of depth. For example: does not identify any strengths/weaknesses, analysis of strengths/weaknesses is too shallow, does not identify any patterns (4.30 ± 0.24) *(Scale 1)*
3	Situation 9 – The mentor tries to build a good rapport with me (4.24 ± 0.09) *Scale 2: Mentor presence*	Situation 20 – The mentee mainly sees the portfolio as an obligation (4.20 ± 0.17) *(No scale)*
4	Situation 3 –The mentor is focused not only on the portfolio, but also on the discussion of personal experiences (4.23 ± 0.09) *Scale 3: Mentor addressing personal issues*	Situation 25 – The mentee has problems with planning and time management (4.00 ± 0.25) *(Scale 3)*
5	Situation 13 – The mentor challenges me to deepen my reflection (4.21 ± 0.09) *Scale 1: Mentor stimulation of reflection*	Situation 11 – The results obtained by the mentee for the progress test are unsatisfactory (4.00 ± 0.24) *(Scale 4)*
6	Situation 7 – During the progress meetings, the mentor provides feed-back about my progress in the competencies and level of reflection (4.13 ± 0.10) *Scale 1: Mentor stimulation of reflection*	Situation 21 – The mentee has difficulty finding the right balance be-tween studies and private life (3.95 ± 0.22) *(Scale 3)*
7	Situation 25 – The mentor discusses my experiences with planning and time management (4.08 ± 0.09) *Scale 3: Mentor addressing personal issues*	Situation 14 – The learning objectives formulated by the mentee lack specificity. For instance, learning objectives remain general and are not grounded in sound reflection on experiences (3.95 ± 0.20) *(Scale 4)*
8	Situation 18 – The mentor encourages open discussion of my personality traits (4.03 ± 0.10) *Scale 3: Mentor addressing personal issues*	Situation 38 – The mentee is insecure about assessment/assessment feedback (3.80 ± 0.27) *(Scale 2)*
9	Situation 12 – The mentor encourages me to reflect on specific personal experiences (4.02 ± 0.10). *Scale 1: Mentor stimulation of reflection*	Situation 18 – The mentee has a distinctive personality (perfectionist, dominant, for example (3.80 ± 0.25) *(Scale 1)*
10	Situation 17 – The mentor encourages open discussion of my personal experiences affecting my studies (4.00 ± 0.10) *Scale 3: Mentor addressing personal issues*	Situation 27 - The mentee expects *(not)* too much of the mentor (is not aware of the limits to the support) (3.80 ± 0.23 > *reversed*) (Not part of scale)
B. It is mentors’ responsibility/ job to take action when this situation occurs (1 = disagree, not the mentors’ responsibility, 3 = sometimes, depends on the situation, 5 = agree, this should always be discussed, the mentor should act). In brackets are the mean ± sem.		
1	Situation 1 - The mentor exhibits professional behaviour (4.75 ± 0.05) *Scale 2: Mentor presence*	Situation 14 - The learning objectives formulated by the mentee lack specificity (4.25 ± 0.16) *(Scale 4)*
2	Situation 7 - During the progress meetings, the mentor provides feedback about my progress in the competencies and level of reflection (4.71 ± 0.05) *Scale 1: Mentor stimulation of reflection*	Situation 13 - Reflections by the mentee fall short of depth (4.20 ± 0.19) *(Scale 1)*
3	Situation 13 - The mentor challenges me to deepen my reflection (4.54 ± 0.06) *Scale 1: Mentor stimulation of reflection*	Situation 6 – The mentee is mainly focused on one or a few key or enabling competencies, which comes at the expense of the discussion of other key or enabling competencies (4.20 ± 0.14) *(Scale 1)*
4	Situation 35 - The mentor is readily available for contact. (4.49 ± 0.06) *Scale 2: Mentor presence*	Situation 8 - The mentee is not reflective. For example: denies his or her own part in certain happenings; keeps stuck in own perspective and his or her self-image is not reflected in the feedback received from others (4.05 ± 0.19) *(Scale 1)*
5	Situation 14 – The mentor challenges me to formulate specific and attainable learning objectives (4.41 ± 0.07) *Scale 4: Mentor stimulating conditions and perspectives for behavioural change*	Situation 5 – The mentee does not treat confidential information obtained in the workplace with care (4.00 ± 0.28) *(Scale 4)*
6	Situation 6 – The mentor pays sufficient attention to the discussion of all key and enabling competencies (4.33 ± 0.08) *Scale 1: Mentor stimulation of reflection*	Situation 1 - The mentee does not exhibit any professional behaviour. For instance: insufficient preparation by mentee of mentor meeting or portfolio; does not keep his/her engagements (4.00 ± 0.16) *(Scale 2)*
7	Situation 26 – The mentor challenges me to avail myself of / use my own qualities, values and strengths (4.30 ± 0.06) *Scale 5: Mentor addressing professional identity development*	Situation 2 – The mentee is little proactive in the workplace (3.85 ± 0.22) *(Scale 3)*
8	Situation 10 – The mentor helps me to formulate learning objectives in competency domains that are rather thorny (4.27 ± 0.08) *Scale 4: Mentor stimulating conditions and perspectives for behavioural change*	Situation 11 - The results obtained by the mentee for the progress test are unsatisfactory (3.85 ± 0.23) *(Scale 4)*
9	Situation 16 – The mentor follows up on the attainment of my predefined learning objectives (4.27 ± 0.07) *Scale 4: Mentor stimulating conditions and perspectives for behavioural change*	Situation 25 - The mentee has problems with planning and time management (3.80 ± 0.23) *(Scale 3)*
10	Situation 28 – The mentor encourages me to reflect on my development (through placements, over the years) (4.25 ± 0.07) *Scale 1: Mentor stimulation of reflection*	Situation 3 - The mentee is focused only on the portfolio, and less on the discussion of personal experiences (3.80 ± 0.21) *(Scale 3)*

The support of behavioural change was resonated in the mentees’ Top 10 of what was expected from the mentor (Table 2b). In the high scoring tasks or expectations of the mentor, Scale 4 was presented more often, as was Scale 5 (Mentor addressing professional identity development). The mentors also indicated that stimulating behavioural change was an important task (Scale 4), next to stimulating reflection (Scale 1) and addressing personal issues (Scale 3). The situations of Scale 2 (Mentee presence, in the perspective of the mentor) were not part of the Top 10 of the mentor, suggesting that mentors put less emphasis on taking responsibility for these situations.

Question 2 of the questionnaire was used to explore which situations were deemed important (question 2), but not experienced (question 1). A large difference between question 2 and question 1 was considered as a gap or a need of the mentee, or a potential obstacle for an effective meeting in the perspective of the mentor (Table 3). From the mentees’ perspective, there was a gap regarding the stimulation of behavioural change (Scale 4) and attention for personal issues (Scale 3) was also presented more often in the mentee Top 10. From the mentors’ perspective, critical situations concerning personal issues (Scale 3) were more often presented. In addition, reflective activities (Scale 1) were mentioned as a potential obstacle for an effective meeting. Item 37 (Mentee list: The mentor is sensitive to my (cultural) background (deleted from Scale 5)) was present in the mentees’ and mentors’ Top 10, indicating that this was a potential situation or critical incident to consider for a needs analysis.

**Table 3:** Gap (difference) between what was experienced (question 1) and deemed important (question 2) – Top 10. In brackets are mean ± sem, and number (n) of mentees/ mentors that experienced the gap

	Mentee (*n* = 103)	Mentor (*n* = 20)
1	Situation 37 – The mentor is sensitive to my (cultural) background (2.06 ± 0.34, *n* = 17). *Not part of a scale*	Situation 35 – The mentee is insufficiently proactive when it comes to contacting the mentor (2.57 ± 0.43, *n* = 7) *(Scale 3)*
2	Situation 29 – The mentor discusses how he or she will treat confidential information about me (1.88 ± 0.14, *n* = 49) *Scale 4: Mentor stimulating conditions and perspectives for behavioural change*	Situation 37 – The mentee is from a different culture which raises difficulties during the meeting (2.31 ± 0.25, *n* = 16) (*Not part of a scale)*
3	Situation 15 – The mentor discusses with me how I can pursue my learning objectives in practice (1.83 ± 0.14, *n* = 48) *Scale 4: Mentor stimulating conditions and perspectives for behavioural change*	Situation 9 – The mentee is insufficiently committed to building a good rapport with the mentor (not open with mentor, does not listen actively during mentor meetings, does not bring forward personal issues to discuss) (2.20 ± 0.37, n = 17) *(Scale 2)*
4	Situation 9 – The mentor tries to build a good rapport with me (1.78 ± 0.25, *n* = 18) *Scale 2: Mentor presence*	Situation 32 – The mentee’s motivation to study fluctuates to a considerable degree (ups and downs) (2.20 ± 0.28, *n* = 5) *(Scale 3)*
5	Situation 24 – The mentor discusses with me my insecurities with respect to the choices I need to make about studies/career (1.74 ± 0.16, *n* = 38) *Scale 3: Mentor addressing personal issues*	Situation 30 – The mentee does not share any thoughts or feelings regarding the meeting with the mentor (2.17 ± 0.27, *n* = 12) *(Scale 4)*
6	Situation 5 – The mentor discusses how I should treat confidential information about the workplace with care (1.72 ± 0.17, *n* = 36) *Scale 4: Mentor stimulating conditions and perspectives for behavioural change*	Situation 12 – The mentee shares little concrete personal experiences with the mentor (2.08 ± 0.21, *n* = 13) *(Scale 1)*
7	Situation 16 – The mentor follows up on the attainment of my predefined learning objectives (1.66 ± 0.14, *n* = 38) *Scale 4: Mentor stimulating conditions and perspectives for behavioural change*	Situation 34 - The mentee is not very proactive when it comes to net-working and forging relevant contacts and relationships for study and career choice-related purposes (2.00 ± 0.58, n = 4) *(Scale 3)*
8	Situation 32 – The mentor pays attention to my incentives to study (e.g., motivational ups and downs) (1.65 ± 0.16, *n* = 26) *Scale 3: Mentor addressing personal issues*	Situation 7 - The mentee is unpleasantly surprised by certain feed-back or an assessment received from the mentor *(*2.00 ± 0.38, *n* = 8*) (Scale 1)*
9	Situation 34 – The mentor supported me in forging new, relevant contacts for study and career choice-related purposes (1.65 ± 0.15, *n* = 49) *Scale 3: Mentor addressing personal issues*	Situation 1 - The mentee does not exhibit any professional behaviour *(*1.90 ± 0.28, *n* = 10*) (Scale 2)*
10	Situation 11 – The mentor discusses and analyses the results of the progress test with me (1.62 ± 0.15, *n* = 24). *Scale 4: Mentor stimulating conditions and perspectives for behavioural change*	Situation 2 - The mentee is little proactive in the workplace *(*1.89 ± 0.31, *n* = 9*) (Scale 3)*

### The dynamic nature of the mentoring process in mentee groups in the different years of the program

The mentoring process is expected to change over time when the mentees further develop and progress in their training. In Table 4a, it is shown that the mentees in different year groups experienced that more emphasis was given by the mentor on stimulation of reflection (Scale 1), the addressing of personal issues (Scale 3) and the stimulation of professional identity development as a medical doctor (Scale 5). Mentor presence (Scale 2) seemed constant, except that the mentor was more available for contact during the years. Importantly, the mentees in different year groups evaluated that these mentoring processes occurred with less guiding of the mentor (Situation 22), suggesting that the mentee was able to better self-direct learning over the years.

**Table 4:** Evaluation in student groups in the different years of the program (Year 1–2-3) of situations that were experienced (Table 4a: Question 1) and perception of the mentors’ tasks or responsibilities (Table 4bs: Question 3). Oneway ANOVA statistical analysis with Bonferroni Post-Hoc tests

Situation	Mean ± sem Year 1	Mean ± sem Year 2	Mean ± sem Year 3	ANOVA *p* value	df	F coef-ficient	Post-hoc Bonferroni testing
A. The situation was experienced in a mentor meeting (1 = never, 5 = at multiple occasions/ repeatedly), as an indication if the situation was (actually) experienced							
SCALE 1: *Mentor stimulation of reflection*							
4. The mentor challenges me to go beyond the bounds of my (sufficient/good) current performance	3.69 ± 0.17	3.77 ± 0.17	4.27 ± 0.13	0.025	102	3.82	Year 1–3 *p* = 0.031
6. The mentor pays sufficient attention to the discussion of all key and enabling competencies	3.51 ± 0.17	3.94 ± 0.16	4.09 ± 0.13	0.022	102	3.96	Year 1–2 *p* = 0.025
8. The mentor encourages me to analyse my self-image critically.	4.03 ± 0.15	3.68 ± 0.18	4.27 ± 0.13	0.032	102	3.55	Year 2–3 *p* = 0.028
28. The mentor encourages me to reflect on my development (through placements, over the years).	3.56 ± 0.18	3.97 ± 0.18	4.48 ± 0.11	0.000	100	8.56	Year 1–3 *p* = 0.0001
SCALE 2: *Mentor presence*							
30. The mentor shares his or her thoughts and feelings about incidents that occur during the mentor meetings.	2.82 ± 0.19	3.27 ± 0.20	3.53 ± 0.21	0.035	100	3.48	Year 1–3 *p* = 0.033
35. The mentor is readily available for contact.	3.46 ± 0.17	4.53 ± 0.13	4.09 ± 0.16	0.000	100	11.73	Year 1–2 p = 0.0001 Year 1–3 *p* = 0.015
SCALE 3: *Mentor addressing personal issues*							
24. The mentor discusses with me my insecurities with respect to the choices I need to make about studies/career.	2.87 ± 0.19	2.97 ± 0.20	3.66 ± 0.21	0.014	99	4.47	Year 1–3 *p* = 0.018
36. The mentor shares his or her experiences with me during meetings.	3.00 ± 0.22	3.83 ± 0.15	3.78 ± 0.21	0.005	100	5.64	Year 1–2 *p* = 0.013 Year 1–3 *p* = 0.019
SCALE 5: *Mentor addressing professional identity development*							
26. The mentor challenges me to avail myself of /use my own qualities, values and strengths.	3.59 ± 0.17	4.10 ± 0.14	4.15 ± 0.16	0.020	102	4.04	Year 1–3 *p* = 0.036
19. The mentor discusses with me how I envision the development of my identity into a doctor	2.67 ± 0.17	3.35 ± 0.16	3.91 ± 0.17	0.000	102	14.16	Year 1–2 p = 0.015 Year 1–3 p = 0.0001
*Not part of a scale*							
22. For the most part of the meetings the mentor is assuming a guiding role (giving suggestions and advice).	3.18 ± 0.16	3.52 ± 0.21	2.76 ± 0.19	0.020	102	4.08	Year 2–3 *p* = 0.016
B. It is mentors’ responsibility/ job to take action when this situation occurs (1 = disagree, not the mentors’ responsibility, 3 = sometimes, depends on the situation, 5 = agree, this should always be discussed, the mentor should act), as an indication of what behaviour or function was perceived as important.							
SCALE 1: *Mentor stimulation of reflection*							
18. The mentor encourages open discussion of my personality traits (such as perfectionist behaviour; dominant behaviour).	4.31 ± 0.13	3.90 ± 0.13	4.39 ± 0.12	0.026	101	3.80	Year 2–3 *p* = 0.034
SCALE 2: *Mentor presence*							
30. The mentor shares his or her thoughts and feelings about incidents that occur during the mentor meetings.	3.13 ± 0.14	3.37 ± 0.16	3.81 ± 0.21	0.016	100	4.34	Year 1–3 p = 0.013
31. The mentor pays attention to my incentives to study (e.g., motivational ups and downs).	3.77 ± 0.15	3.67 ± 0.18	4.28 ± 0.16	0.024	100	3.87	Year 2–3 *p* = 0.037
36. The mentor shares his or her experiences with me during meetings	2.85 ± 0.20	3.47 ± 0.18	3.53 ± 0.22	0.027	100	3.75	Year 1–3 *p* = 0.048
SCALE 3: *Mentor addressing personal issues*							
3. The mentor is focused not only on the portfolio, but also on the discussion of personal experiences	4.23 ± 0.12	3.45 ± 0.19	4.33 ± 0.14	0.000	102	10.13	Year 1–2 *p* = 0.001 Year 2–3 p = 0.0001
21. The mentor discusses the balance between my studies and private life with me	3.85 ± 0.16	3.68 ± 0.18	4.42 ± 0.12	0.003	102	6.13	Year 1–3 *p* = 0.024 Year 2–3 *p* = 0.004
SCALE 4: *Mentor stimulating conditions and perspectives for behavioural change*							
5. The mentor discusses how I should treat confidential information about the workplace with care	2.95 ± 0.19	2.67 ± 0.19	2.12 ± 0.21	0.011	100	4.70	Year 1–3 *p* = 0.009
SCALE 5: *Mentor addressing professional identity development*							
19. The mentor discusses with me how I envision the development of my identity into a doctor.	3.69 ± 0.17	3.74 ± 0.17	4.33 ± 0.13	0.009	102	4.93	Year 1–3 *p* = 0.014 Year 2–3 *p* = 0.039
26. The mentor challenges me to avail myself of / use my own qualities, values and strengths.	4.13 ± 0.11	4.26 ± 0.12	4.55 ± 0.10	0.022	102	3.95	Year 1–3 *p* = 0.020
*Not part of a scale*							
22. For the most part of the meetings the mentor is assuming a guiding role (giving suggestions and advice).	3.41 ± 0.17	3.55 ± 0.20	2.70 ± 0.19	0.004	102	5.94	Year 1–3 p = 0.018 Year 2–3 *p* = 0.006

What the mentees of different year groups evaluated resonated partly with what was expected from the mentor during the program (Table 4b). It was clear that addressing professional identity development was deemed an important task of the mentor during the years (Scale 5). The noted tempering of coaching was expected (item 22), suggesting that the mentees needed less coaching. In addition, and this was less evident in what was evaluated (Table 4a), mentees in different year groups expected their mentor to be more personal (Scale 2, Mentor presence), such as sharing their thoughts and addressing their own experiences.

Comparing the evaluations of mentees in different year groups of what was expected of the mentor (Table 4b) with the apparent need or gap (Table 3) suggested that the discussion of confidential information was perceived as a gap (Scale 4, situation 5, Table 3) by the mentees, but the mentees perceived it less as the task of the mentor during the course of the program (Table 4b). This was considered an inconsistency and was a signal that this needed to be addressed in either the training of students or faculty development of mentors.

## Discussion

In this study, a dual-purpose questionnaire was designed and validated that enabled the exploration of both the mentor and mentee perceptions of the mentoring process and its critical incidents. PCA showed that specific sets of questions addressed important elements in the mentoring process, such as self-direction of learning and reflection (Scale 1), guidance of behavioural change (Scale 4), addressing personal issues and professional identity development (Scale 3 and 5) and how the mentor and mentee presents oneself in the mentoring relationship (Scale 2) *(Research question 1)*.

Mentors and mentees perceived comparable situations as critical or important for an effective mentoring process, although in the mentors’ perspective the items of Scale 4 (Mentor stimulating conditions and perspectives for behavioural change) had relatively higher scores, showing that there were nuances in what mentees and mentors find important or critical. In addition, the comparison of what was experienced (questionnaire question 1) and what was expected (questionnaire question 2) showed gaps in the mentoring practices in the context of this program, such as (lack of) attention for cultural issues (mentee perspective), resonating with the mentor perception that cultural issues were considered challenging *(Research question 2)*. By comparison of the mentee groups in the different years of the program, the dynamic or evolving nature of the mentor process became evident, mentees experienced more emphasis by the mentor on reflection (Scale 1), at a constant level of mentor presence (Scale 2) *(Research question 3)*.

In the current study, as a proof of concept, the questionnaire was completed by the mentees and mentors of a medical program, to assess its suitability for a needs analysis and to determine whether use of the questionnaire would demonstrate gaps or misalignments in the mentoring program. As widely acknowledged, mentoring is an individualized, context-specific and organizational-dependent process [15, 18] and there has been calls for evidence-based methods to study mentoring approaches [18]. However, there is also a need for the possibility to tailor methods given the variances in mentoring practice. In terms of evidence-based, the questionnaire was designed on the basis of qualitative data [12] and theory and research about mentoring. In addition, analysis showed that the scales of the questionnaire addressed important, well-established elements in mentoring processes, such as self-regulation of learning, coaching of behavioural change and personal development [1, 2, 5, 11, 15, 27, 29]. In particular, the scale of mentor presence resonates with the themes of teacher authenticity, for which learners emphasized expertise, passion, unicity and distance [35] and the theme of presence, for which teachers emphasize presence as self-awareness, as connection to students, and as connection to subject matter [36]. This is indeed an equilibrium that mentors need to maintain. Also, the theme of professional identity formation is essential in medical education, which was shown to be relational and social in nature, with mentors and experiential learning as important educational strategies [37]. The alignment of the scales with theory and research would make the questionnaire suitable to use in other contexts given that the goal of mentoring is self-direction of learning and there is a longitudinal element, a longer-lasting mentoring relationship. In our context, the open-ended question at the end of the questionnaire showed that mentees and mentors missed items on a context-specific procedure, i.e. a second mentor being present at 1 of the meetings, giving feedback on progress and use of the portfolio [12]. In other contexts, elements not addressed in the questionnaire could be monitored by either additional questions in a follow-up questionnaire or tailored methods such as short interviews to complement the need analysis of the questionnaire.

The questionnaire was specifically designed with the dual purpose to capture the perceptions and expectations of both the mentee and the mentor. The data and information can be used at multiple levels. Firstly, by the program or organisation to evaluate the mentoring system, e.g. does the mentoring system achieve the desired goal? Analysis of the results could lead to either tailored faculty development training, e.g. use of the critical incidents (role-played in video-vignettes) in the training of mentors, emphasis of certain information in guidelines, or a change in the procedures, in order to (further) improve the quality of the mentoring system. There are limited studies on the efficacy and usefulness of a formal evaluation process of the mentoring system [18, 38]. Sng et al. [18] emphasized that there is a need to evaluate the development of mentoring processes and the impact on the mentor-mentee relationship. Standardized, validated measurement tools or questionnaires could be helpful for this. The second level is at the individual mentor, release of the results of the individual mentors’ group of mentees (anonymized) can inform the mentor if there is a gap or discrepancy in 1 of the scales or if there is a need to work on or get extra training in certain mentoring competencies [30]. Fleming et al. [30] designed a measurement tool to assess mentor competencies in the context of research mentors, which were categorised as maintaining effective communication, aligning expectations, assessing understanding, addressing diversity, fostering independence, and promoting professional development. These competencies are likely to be more generic and also applicable in the educational field. With the exception of addressing diversity, these could be recognized in Scale 2, 3, 4 and 5 of the questionnaire. A third level is the data that can be evaluated in the different years of the program, applicable both at the level of the organisation and the individual mentor. As shown, the mentees valued some aspects more in the different years of the program, or needed less of certain mentoring functions. The role of the mentor clearly changed, students became more self-directed. Mentors need to be aware that this needs another level of guidance or emphasis on other mentor competencies. Mentor training or faculty development for mentors is a valuable tool. It is well known that mentor training leads to more confident mentors and improved mentoring skills also in the perception of the mentees [39, 40]. For the program in the current study, analysis of the data of the questionnaire led to a dialogue in the mentor training about the topic of confidentiality of documents that are used in the portfolio, and how to address cultural diversity. In addition, video-vignettes of a mentor-meeting were made, in which the high score critical situations were discussed or the topic of the mentor-mentee dialogue. These vignettes are currently used in the training sessions.

The findings of this study should be interpreted given certain limitations. Firstly, this is an initial validation of the questionnaire. The sample size for a component analysis should have a ratio of 10 participants per item and that number (380) was not reached. Additional studies with larger number of respondents are needed to confirm these first results. Secondly, the components were only identified for the mentees; mentors could have a different representations of the dimensions evaluated in the questionnaire. Thirdly, this study was performed in one institute, and the questionnaire captured a number of context-specific elements, such as the progress test. In addition, the portfolio was assesses at the end of each year, which might have had an effect on the mentoring relationship. Although the mentors are not assessors in our setting and care is taken to strictly divide the mentoring and assessing role, the high-stake context of the portfolio assessment at the end of the year and the use of programmatic assessment are factors that may have affected the results of the questionnaire in this study. Therefore, the results may not be transferable to other settings and if the questionnaire is used in other settings, it should be adapted to the specific context. Nevertheless, the use of a longitudinal, portfolio-based mentoring system is becoming more common in medical education, so the results might be of interest to other programs. Fourthly, although the response rate was considered acceptable (> 60% [41]), the potential difference in non-responders versus responders should be considered. The non-responders were mentees or mentors that were not present at the plenary session where the questionnaire was distributed and collected. In this medical course, these sessions are usually attended by 60–70% of the population. As the questionnaire was anonymous, we could not follow-up on the non-responders. Although it is unlikely that these represent a certain sub-population, we cannot fully eliminate that this may have affected the results. Finally, further development is needed for the use of the questionnaire in an international, non-western context, to address cultural diversity.

## Conclusions

In this study a dual purpose questionnaire was designed and validated to capture both the mentor and mentee perceptions of the mentoring process and its critical incidents. The questionnaire contained 5 scales that could be associated to important processes in the mentoring practice and mentor competencies (*Research question 1*). Analysis of the data of the questionnaire disclosed a number of gaps or misalignments in the mentors’ and mentees’ experiences and expectations in a medical program (*Research question 2*). Use of the questionnaire during the course of the program showed that the expectations of the mentees on the mentoring process changed (*Research question 3*), again disclosing the need to critically examine the mentoring system and attune the mentor training program. Given the individualized, context-specific, dynamic and organizational-dependent nature of mentoring practices, programs would benefit from a regular evaluation of mentoring practices, in order to facilitate organizational revisions of the mentoring system and further development of the mentoring competencies of individual mentors.

## Abbreviations

PCA: Principal Component Analysis
sem: Standard error of the mean
